# Segregated cation flux by TPC2 biases Ca^2+^ signaling through lysosomes

**DOI:** 10.1038/s41467-022-31959-0

**Published:** 2022-08-02

**Authors:** Yu Yuan, Dawid Jaślan, Taufiq Rahman, Stephen R. Bolsover, Vikas Arige, Larry E. Wagner, Carla Abrahamian, Rachel Tang, Marco Keller, Jonas Hartmann, Anna S. Rosato, Eva-Maria Weiden, Franz Bracher, David I. Yule, Christian Grimm, Sandip Patel

**Affiliations:** 1 Department of Cell and Developmental Biology, University College London, London, UK; 2 Walther Straub Institute of Pharmacology and Toxicology, Faculty of Medicine, Ludwig-Maximilians University, Munich, Germany; 3 Department of Pharmacology, University of Cambridge, Cambridge, UK; 4 Department of Pharmacology and Physiology, University of Rochester, Rochester, NY USA; 5 Department of Pharmacy—Center for Drug Research, Ludwig-Maximilians University, Munich, Germany

**Keywords:** Calcium signalling, Patch clamp, Lysosomes, Ion channel signalling

## Abstract

Two-pore channels are endo-lysosomal cation channels with malleable selectivity filters that drive endocytic ion flux and membrane traffic. Here we show that TPC2 can differentially regulate its cation permeability when co-activated by its endogenous ligands, NAADP and PI(3,5)P_2_. Whereas NAADP rendered the channel Ca^2+^-permeable and PI(3,5)P_2_ rendered the channel Na^+^-selective, a combination of the two increased Ca^2+^ but not Na^+^ flux. Mechanistically, this was due to an increase in Ca^2+^ permeability independent of changes in ion selectivity. Functionally, we show that cell permeable NAADP and PI(3,5)P_2_ mimetics synergistically activate native TPC2 channels in live cells, globalizing cytosolic Ca^2+^ signals and regulating lysosomal pH and motility. Our data reveal that flux of different ions through the same pore can be independently controlled and identify TPC2 as a likely coincidence detector that optimizes lysosomal Ca^2+^ signaling.

## Abstract (web summary)

TPC2 is a lysosomal ion channel permeable to both calcium and sodium ions. Here, the authors show that TPC2 can selectively increase its calcium permeability when simultaneously challenged by both its natural activators- NAADP and PI(3,5)P_2_.

## Introduction

Sensing signals and coordinating the ensuing outputs is vital for maintaining cell and tissue homeostasis. To this end, cells possess a battery of ion channels on both the plasmalemma and in organelles that open in response to specific cues. It is clear now that the lysosome, traditionally viewed as the cell’s recycling center, is a signaling organelle endowed with a number of ion channels linked to diseases^1–3^. Understanding how these channels are regulated is vital to understand cell function and dysfunction.

Two-pore channels (TPCs) are a class of evolutionarily ancient, ubiquitously expressed ion channels that localize to lysosomes and other acidic organelles in animal cells^4–6^. Here, they regulate a diverse range of processes including both vesicular^7^ and non-vesicular^8^ membrane traffic. They are fast emerging as drug targets in disorders such as viral infection^9,10^ and cancer^11^. But despite such considerable patho-physiological importance, their activation mechanisms are ill-defined. On the one hand, they are described as Ca^2+^-permeable channels activated by NAADP^12–16^. NAADP is a water soluble Ca^2+^ mobilizing messenger that triggers Ca^2+^ release primarily from acidic organelles to regulate numerous Ca^2+^-dependent outputs^17,18^. But on the other hand, TPCs are described as Na^+^ channels activated by PI(3,5)P_2_^19–21^. PI(3,5)P_2_ is a minor, endo-lysosomal-enriched phosphoinositide produced by the PIKfyve complex that regulates organelle size, autophagy and endocytic membrane traffic^22,23^.

Our recent work showed that the ion selectivity of TPC2 is not fixed, as is generally assumed for ion channels, but rather agonist-dependent^24^. This unique property reconciles contradictory findings relating to gating and ionic permeability of TPC2. Discovery of lipophilic TPC2 agonists revealed that one of these molecules rendered the channel more Ca^2+^-permeable mimicking the effect of NAADP whereas the other rendered the channel more Na^+^-selective mimicking the effect of PI(3,5)P_2_^24^. These agonists also revealed distinct effects on lysosomal activity in cells introducing a paradigm whereby the same ion channel can mediate unique cellular outputs through distinct ion fluxes. This raises the question of how TPC2 behaves under physiological conditions when it is simultaneously exposed to conflicting endogenous cues.

Our results show that the Ca^2+^ but not Na^+^ permeability of TPC2 is selectively enhanced when the channel is co-activated by its ligands. Ca^2+^ and Na^+^ flux by TPC2 can therefore be independently controlled. Such regulation translates into robust global Ca^2+^ signals in a number of cell types but not in TPC2 knockout cells, impacting lysosomal activity in a synergistic way. We suggest TPC2 as a functional coincidence detector that tunes its ionic behavior on demand to suit signaling needs.

## Results

### TPC2 agonists synergistically activate TPC2

**Fig. 1:**
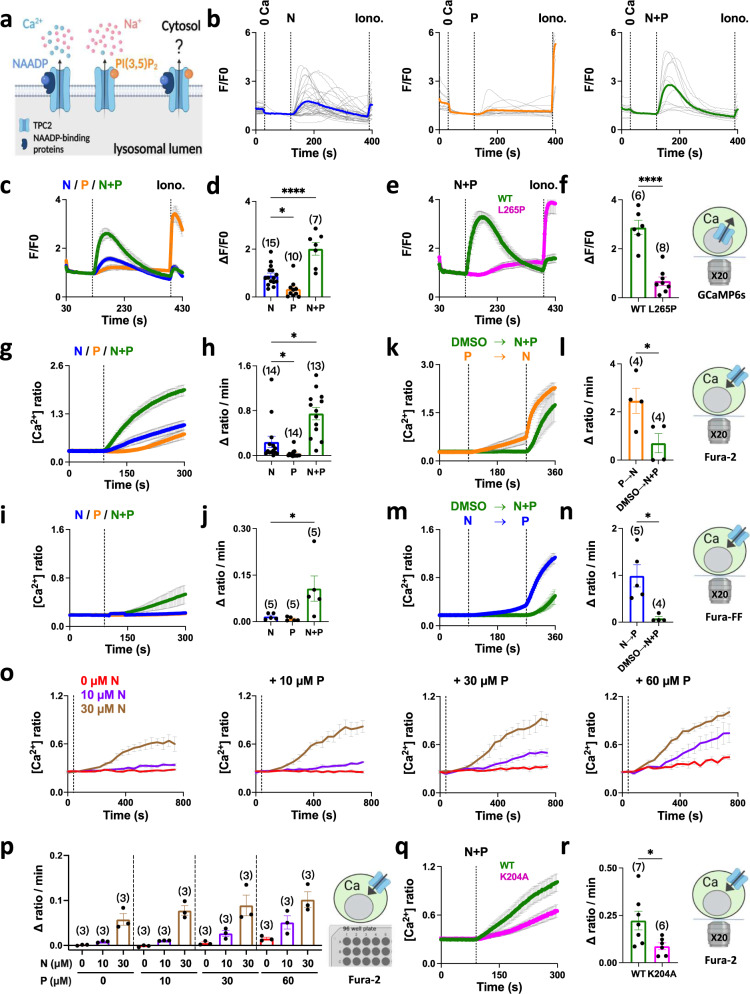
TPC2 agonists synergistically activate TPC2. **a** Differential effects of TPC2 activators on cation flux. **b**–**d** Effect of TPC2-A1-N (N; 30 µM), TPC2-A1-P (P; 60 µM) or a combination of the two (N+P) on Ca^2+^ levels of individual HeLa cells transiently transfected with TPC2 fused to GCaMP6s. Each trace in **b** is the fluorescence response of a single cell imaged from a typical field of view. The thicker trace is the average of the population. External Ca^2+^ was removed (0 Ca) prior to stimulation and ionomycin (iono., 2 µM) added at the end of the experiment. **c** pooled time-course data expressed as mean ± s.e.m. from 7 to 15 experiments. **d** peak change in signal from multiple experiments where each point represents the mean response of all cells from an independent experiment. *p = 0.01, ****p < 0.0001 (One-way ANOVA followed by Dunnett’s post hoc test). **e**, **f** Effect of the TPC2-A1-N (30 µM) and TPC2-A1-P (60 µM) combination on Ca^2+^ levels of wild type (WT) or pore-dead TPC2 (L265P) fused to GCaMP6s. Pooled data expressed as mean ± s.e.m. from 6 to 8 experiments (**e**) and the peak change in signal from multiple experiments (**f**). ****p < 0.0001 (Unpaired t-test, two-tailed). **g**–**j** Effect of TPC2-A1-N (30 µM), TPC2-A1-P (60 µM) or a combination of the two on Ca^2+^ levels of HEK cells stably expressing TPC2^L11A/L12A^. Cells were loaded with Fura-2 (**g**, **h**) or Fura-FF (**i**, **j**). Data are expressed as time-courses (mean ± s.e.m. from 5 to 14 experiments; **g**, **i**) and the rate of Ca^2+^ entry (**h**, **j**). *p < 0.05 (Kruskal-Wallis test followed by Dunn’s post hoc test) (**h**); *p = 0.03 (Mann-Whitney test, two-tailed) (**j**). **k**–**n** Effect of sequential agonist additions on Ca^2+^ levels in HEK cells stably expressing TPC2^L11A/L12A^. In **k**, Fura-2-loaded cells were stimulated with TPC2-A1-N (30 µM) after TPC2-A1-P (60 µM) and the response (mean ± sem from 4 experiments) compared that when the agonists were added simultaneously. In **m**, Fura-FF-loaded cells were stimulated with TPC2-A1-P (60 µM) after TPC2-A1-N (30 µM) (mean ± sem from 4 to 5 experiments). Pooled data quantifying the rate of Ca^2+^ entry are shown in **l** and **n**. *p = 0.04 (Unpaired t-test, two-tailed) (**l**); *p = 0.01 (Unpaired t-test, two-tailed) (**n**). **o**–**p** Effect of increasing concentrations of TPC2 agonists on Ca^2+^ levels in Fura-2-loaded HEK cells stably expressing TPC2^L11A/L12A^. Cells were stimulated with 10 and 30 µM TPC2-A1-N in the presence of 10, 30 and 60 µM TPC2-A1-P using an automated plate reader. Data are expressed as mean ± s.e.m. from 3 experiments (**o**) and the rate of Ca^2+^ entry (**p**). **q**–**r** Effect of the TPC2-A1-N (10 µM) and TPC2-A1-P combination (30 µM) on Ca^2+^ levels of Fura-2-loaded HeLa cells transiently expressing cell surface TPC2 (TPC2^L11A/L12A^) or a lipid-dead mutant (TPC2^L11A/L12A/K204A^). Pooled data expressed as mean ± sem from 6 to 7 experiments (**q**) and the rate of Ca^2+^ entry (**r**). *p = 0.04 (Unpaired t-test, two-tailed). Source data are provided as a Source Data file.

NAADP and PI(3,5)P_2_ have dramatically different effects on TPC2 rendering the channel either more Ca^2+^-permeable or more Na^+^-selective^24^. What happens when the channel is co-activated (Fig. 1a)? To answer this, we first used cells expressing the genetically-encoded Ca^2+^ indicator GCaMP6s fused to the cytosolic C-terminus of TPC2 to record release of Ca^2+^ into the cytosol. Stimulation of these cells with the cell-permeable NAADP mimetic TPC2-A1-N evoked a readily recordable Ca^2+^ response whereas the PI(3,5)P_2_ mimetic TPC2-A1-P induced only a minor one (Fig. 1b). Surprisingly, co-addition of the agonists evoked a markedly potentiated Ca^2+^ response (Fig. 1b–d). This effect was dependent on TPC2 containing a functional pore because little Ca^2+^ release could be detected in parallel experiments using cells expressing a ‘pore-dead’ mutant, TPC2^L265P^ (Fig. 1e, f) which substantially reduces (>10-fold) but does not eliminate conductance^25^.

Because release of lysosomal Ca^2+^ results in secondary release from the ER^26^, we also examined the effects of co-stimulating TPC2 in cells stably expressing TPC2 targeted to the plasma membrane (TPC2^L11A/L12A^). In this format, TPC2 behaves as an influx channel uncoupled from ER Ca^2+^ release^25^. Figure 1g, h compares Ca^2+^ signals in response to TPC2-A1-N and TPC2-A1-P alone and in combination in cells loaded with the Ca^2+^-indicator Fura-2. This analysis revealed that the TPC2-A1-P response was characteristically delayed. Strikingly, when the agonists were co-applied to cells, the Ca^2+^ signals were markedly accelerated (Fig. 1g). These data were quantified by measuring the initial rate of Ca^2+^ influx. As summarized in Fig. 1h, there was little influx in response to TPC2-A1-P whereas that of the combination was ~4-fold increased relative to TPC2-A1-N. We also performed experiments with TPC2^L11A/L12A^-expressing cells loaded with the low affinity Ca^2+^ indicator Fura-2 FF (Fig. 1i, j). With this dye, there was little detectable influx over the first 5 min when cells were stimulated with TPC2-A1-N or TPC2-A1-P (Fig. 1i) in accord with its higher K_d_ for Ca^2+^ (5.5 µM) relative to Fura-2 (0.14 µM). But there was substantial influx in response to the agonist combination (Fig. 1i, j) thereby again revealing marked synergism.

To further characterize the effect of the agonist combination, we performed sequential additions. We stimulated TPC2^L11A/L12A^ with TPC2-A1-N after TPC2-A1-P to mimic receptor-mediated signaling events where NAADP levels demonstrably rise^27^. As shown in Fig. 1k, TPC2-A1-N induced robust Ca^2+^ influx. This signal was significantly faster than the combination applied simultaneously (Fig. 1l). Similar results were obtained when the order was reversed. In these experiments, we used Fura-FF to prevent confounding issues of elevated baselines (due to TPC2-A1-N-mediated Ca^2+^ influx) in the quantification of subsequent entry. Under these conditions, TPC2-A1-P again markedly increased Ca^2+^ influx following TPC2-A1-N stimulation over and above that of the combination applied simultaneously (Fig. 1m, n).

Figure 1o shows the results of automated plate reading where the effect of systematically increasing the concentration of TPC2-A1-P on the Ca^2+^ responses to increasing concentrations of TPC2-A1-N was performed. This analysis summarized in Fig. 1p reveals that synergism is concentration-dependent and saturable.

Because the binding site for TPC2-A1-P likely overlaps with that of PI(3,5)P_2_^24^, we compared agonist combination responses in cells transiently expressing a ‘lipid-dead’ mutant, TPC2^K204^. As shown in Fig. 1q–r, Ca^2+^ influx was significantly reduced by the mutation.

In sum, multiple lines of evidence indicate that TPC2 agonists directly activate Ca^2+^ flux through TPC2 in a synergistic way.

### Cation permeability of TPC2 is independently regulated

**Fig. 2:**
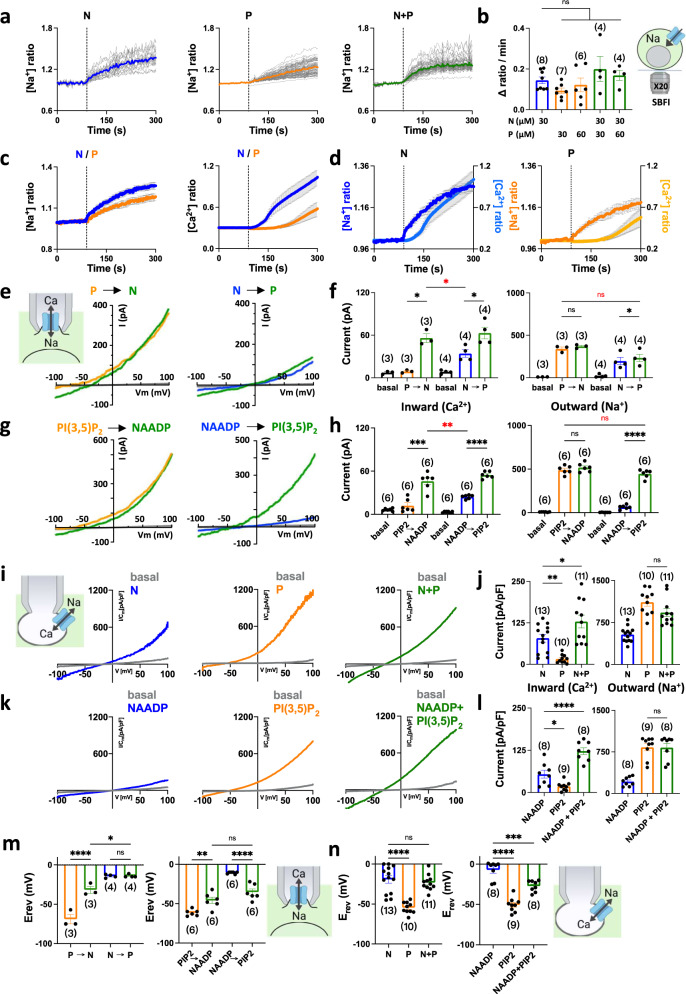
Cation permeability of TPC2 is independently regulated. **a** Effect of TPC2-A1-N (N; 30 µM), TPC2-A1-P (P; 30 µM) or a combination of the two (N+P) on Na^+^ levels of individual SBFI-loaded HEK cells stably expressing TPC2^L11A/L12A^. Each trace is the normalised fluorescence ratio response of a single cell imaged from a typical field of view. The thicker trace is the average of the population. **b** Pooled data (mean ± sem) quantifying the rate of Na^+^ influx from 4-8 experiments in response to the indicated concentration of agonists. n.s. not significant (One-way ANOVA followed by Dunnett’s post hoc test). **c**, **d** Comparison of TPC2-A1-N and TPC2-A1-P responses on cytosolic Na^+^ and Ca^2+^ in TPC2^L11A/L12A^-expressing cells loaded with SBFI and Fura-2, respectively. Data are mean ± sem from 4 to 10 experiments. **e**–**h** Effect of sequential agonist additions on currents from HEK cells stably expressing TPC2^L11A/L12A^ under bi-ionic conditions. Macropatches were stimulated with 10 µM TPC2-A1-N and 10 µM TPC2-A1-P (**e**) or 1 µM PI(3,5)P_2_ and 100 nM NAADP (**g**) in the indicated order. Pooled data (mean ± sem) quantifying the inward Ca^2+^ currents at −100 mV and outward Na^+^ currents at +100 mV from 3 to 6 experiments before (basal) and after agonist addition are shown in **f** and **h**. *p < 0.05, ***p = 0.0003, ****p < 0.0001, n.s. not significant (Paired t-test, two-tailed); *p = 0.04, **p = 0.002, n.s. not significant (Unpaired t-test, two-tailed, in red). **i**–**l** Effect of agonist additions on currents from HEK cells stably expressing TPC2 in lysosomes under bi-ionic conditions. Cells were stimulated with 10 µM TPC2-A1-P or 10 µM TPC2-A1-N (**i**) or 1 µM PI(3,5)P_2_ and 100 nM NAADP (**k**) either alone or in combination. Pooled data (mean ± sem) quantifying the inward Ca^2+^ currents at −60 mV or outward Na^+^ currents at +100 mV from 8 to 13 experiments in response to the agonists are shown in **j** and **l**, respectively. *p = 0.02, **p = 0.005 (One-way ANOVA followed by Dunnett’s post hoc test) (**j**); *p = 0.02, ****p < 0.0001 (One-way ANOVA followed by Dunnett’s post hoc test) (**l**); n.s. not significant (Unpaired t-test, two-tailed, **j** and **l**). **m**, **n** Effect of TPC2 agonists on reversal potentials. Pooled data (mean ± sem from 3 to 13 experiments) quantifying the effect of TPC2 agonists on E_rev_ in HEK cells stably expressing TPC2^L11A/L12A^ at the cell surface (**m**) or TPC2 in lysosomes (**n**). Values were derived from the bi-ionic experiments described in **e**–**l**. *p = 0.01, **p = 0.007, ****p < 0.0001, n.s. not significant (One-way ANOVA followed by Tukey’s post hoc test) (**m**); ***p = 0.0005, ****p < 0.0001, n.s. not significant (One-way ANOVA followed by Dunnett’s post hoc test) (**n**). Source data are provided as a Source Data file.

To further probe the properties of co-activated TPC2, we examined the effects of the agonists on Na^+^ fluxes. To do this, we measured cytosolic Na^+^ using the ratiometric Na^+^ indicator SBFI in cells stably expressing TPC2^L11A/L12A^. As shown in Fig. 2a, b, TPC2-A1-N and TPC2-A1-P (both at 30 µM) induced Na^+^ signals. These signals were reduced upon replacement of extracellular Na^+^ with NMDG (Supplementary Fig. 1a–d) and in cells transiently expressing pore-dead TPC2 at the cell surface (Supplementary Fig. 1e–h) consistent with TPC2-mediated Na^+^ influx. Figure 2c, d compares the Na^+^ and Ca^2+^ signals evoked by each agonist. The kinetics and the amplitude of the Na^+^ signals evoked by TPC2-A1-N and TPC2-A1-P were similar and thus in marked contrast to the Ca^2+^ signals where TPC2-A1-N evoked a more rapid response (Fig. 2c). The absolute rates of Na^+^ and Ca^2+^ influx were therefore more similar for TPC2-A1-N than TPC2-A1-P (Fig. 2d). Strikingly, co-addition of the agonists did not affect the Na^+^ signals at two different combinations (Fig. 2a, b).

The differential effects of the agonist combination on Ca^2+^ and Na^+^ influx raised the intriguing possibility that TPC2 selectively alters its Ca^2+^ permeability upon co-stimulation. To test this directly, we performed macro-patch recording of agonist-evoked currents from cells stably expressing TPC2^L11A/L12A^ under bi-ionic conditions using Ca^2+^ in the pipette solution (extracellular/luminal face of TPC2) and Na^+^ in the bath (cytosolic face of TPC2). As shown in Fig. 2e, TPC2-A1-N induced an inward Ca^2+^ current and an outward Na^+^ current. So too did TPC2-A1-P but the Ca^2+^ current was negligible (Fig. 2e, f). Stimulation of TPC2^L11A/L12A^ with TPC2-A1-N after TPC2-A1-P induced a significant increase in Ca^2+^ current relative to TPC2-A1-N alone (Fig. 2e, f) consistent with enhanced Ca^2+^ signals (Fig. 1). But it had little effect on the Na^+^ current (Fig. 2e, f). Essentially similar results were obtained when the order of the additions was reversed (Fig. 2e, f). Thus, stimulation of TPC2^L11A/L12A^ with TPC2-A1-P after TPC2-A1-N induced a larger Ca^2+^ current but the Na^+^ current was unchanged and smaller than TPC2-A1-P alone (Fig. 2f).

We compared the actions of the synthetic agonists with their endogenous counterparts. As shown in Fig. 2e–h, NAADP induced Ca^2+^ and Na^+^ currents similar to TPC2-A1-N (hit rate 6/10 patches). Like TPC2-A1-P, PI(3,5)P_2_ induced Na^+^ currents only (Fig. 2e–h). Addition of NAADP after PI(3,5)P_2_ or PI(3,5)P_2_ after NAADP resulted in a Ca^2+^ current ~ 2-fold larger than NAADP alone (Fig. 2h). Na^+^ currents in the presence of the combination were not different to PI(3,5)P_2_ alone (Fig. 2h) again suggesting differential regulation of Na^+^ and Ca^2+^ currents by the agonist combination.

Cell surface targeted TPC2 may not faithfully recapitulate the properties of TPC2 in its native environment. We therefore also analyzed TPC2 currents from enlarged lysosomes using vacuolar patch clamp in cells stably expressing TPC2. As shown in Fig. 2i, j, simultaneous addition of TPC2-A1-N and TPC2-A1-P induced a larger Ca^2+^ current than TPC2-A1-N alone. Similar results were obtained when the effects of NAADP and PI(3,5)P_2_ were compared with NAADP alone (hit rate 8/12 patches; Fig. 2k–l). In marked contrast, the Na^+^ currents induced by the synthetic or natural agonist combination were comparable to currents induced respectively by TPC2-A1-P or PI(3,5)P_2_ alone (Fig. 2j, l).

To understand this selective potentiation, we analyzed reversal potentials (E_rev_) to infer the relative permeability of TPC2 to Ca^2+^ and Na^+^ upon agonist stimulation (Fig. 2m, n). E_rev_ for currents mediated by TPC2^L11A/L12A^ in response to TPC2-A1-N and NAADP were similar (~ −10 mV) but more positive than TPC2-A1-P and PI(3,5)P_2_ (~−70 mV) (Fig. 2m). These values correspond to *P*_Ca_/*P*_Na_ values of ~0.6 and ~0.04, respectively. Similar values were obtained for TPC2 expressed in lysosomes (Fig. 2n). These data confirm that both cell surface and lysosomally-targeted TPC2 toggles its ion selectivity between a relatively non-selective state to a more Na^+^-selective one.

E_rev_ increased when TPC2^L11A/L12A^ was challenged with TPC2-A1-N after TPC2-A1-P or with NAADP after PI(3,5)P_2_ (Fig. 2m) consistent with the increased Ca^2+^-current (Fig. 2f, h). However, in both stimulation scenarios, the E_rev_ for the combinations did not reach that for the singletons and instead was intermediate (Fig. 2m). These measured values (~−30–45 mV) corresponded to a *P*_Ca_/*P*_Na_ of ~0.2–0.3 i.e. a moderately Na^+^-selective state. These data indicate that a change in ion selectivity cannot account for the increased Ca^2+^ current obtained in the presence of both agonists. This was even more apparent when the order of stimulations was reversed. Thus, activation of TPC2^L11A/L12A^ with TPC2-A1-P after TPC2-A1-N failed to affect E_rev_ (Fig. 2m) despite a doubling of the Ca^2+^ current (Fig. 2f). Notably, E_rev_ for PI(3,5)P_2_ after NAADP did change adopting an intermediate value similar to NAADP after PI(3,5)P_2_ (Fig. 2m). These data reveal a ‘dominant’ effect of TPC2-A1-N on ion selectivity distinguishing it from NAADP.

Essentially, similar results were found for TPC2 recorded from lysosomes. Thus, E_rev_ for the synthetic agonist combination was not different to TPC2-A1-N alone (Fig. 2n). And E_rev_ for the natural agonist combination was intermediate between NAADP and PI(3,5)P_2_ (Fig. 2n).

Taken together, these data show that upon co-stimulation, TPC2 alters its permeability to Ca^2+^ but not Na^+^ independent of changes in ion selection.

### Co-activation of native TPC2 evokes global Ca^2+^ signals

**Fig. 3:**
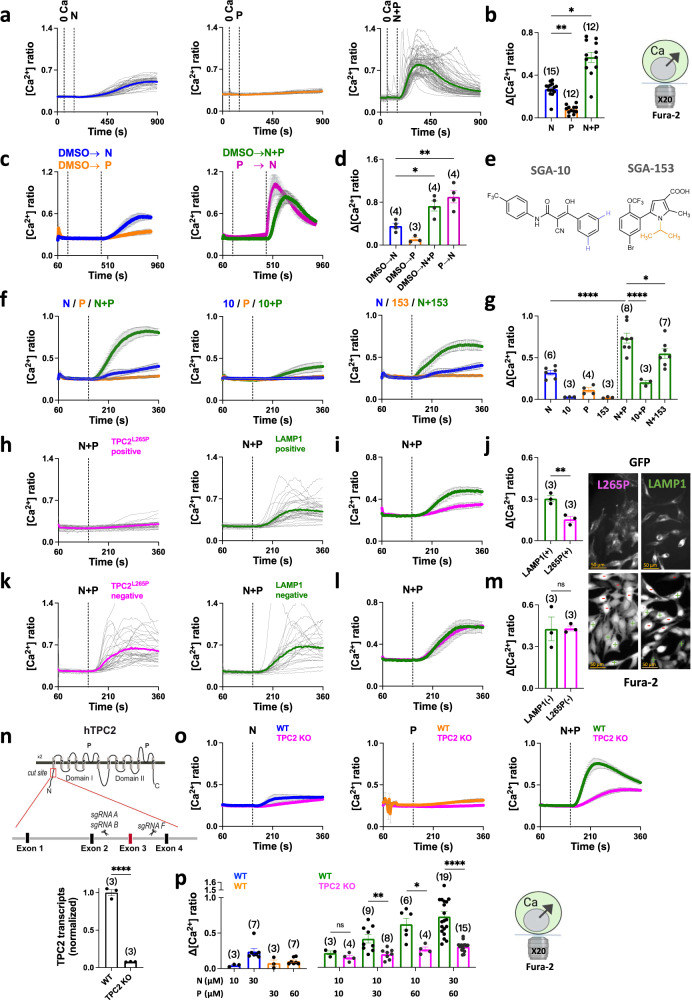
Co-activation of endogenous TPC2 evokes global Ca^2+^ signals. **a**, **b** Effect of TPC2-A1-N (N; 30 µM), TPC2-A1-P (P; 60 µM) or a combination of the two (N+P). on Ca^2+^ levels of individual naïve (untransfected) HeLa cells loaded with Fura-2. Each trace is the fluorescence ratio response of a single cell imaged from a typical field of view (**a**). The thicker trace is the average of the population. External Ca^2+^ was removed (0 Ca) prior to stimulation. Pooled data (mean ± sem) quantifying the peak change in ratio from 12 to 15 experiments where each point represents the mean response of all cells from an independent experiment, are shown in **b**. *p = 0.02, **p = 0.004 (Kruskal-Wallis test followed by Dunn’s post hoc test). **c**, **d** Effect of the agonist combination on Ca^2+^ levels where the agonists were added simultaneously (N+P) or when TPC2-A1-N was added after TPC2-A1-P (P > N) **c**. Data are mean ± sem from 3 to 4 independent experiments. Pooled data quantifying the peak change in ratio is shown in **d**. *p = 0.03, **p = 0.004 (One-way ANOVA followed by Dunnett’s post hoc test). **e** Structures of the inactive TPC2-A1-N analogue, SGA-10 and the inactive TPC2-A1-P analogue, SGA-153. **f**, **g** Effect of SGA-10 (10; 30 µM) and SGA-153 (153, 60 µM) on Ca^2+^ levels. Cells were co-stimulated with TPC2-A1-N or TPC2-A1-P as indicated. Pooled data (mean ± sem from 3 to 8 experiments) quantifying the peak change in ratio is shown in **g**. *p = 0.04, ****p < 0.0001 (One-way ANOVA followed by Dunnett’s post hoc test). **h**–**m** Effect of TPC2^L265P^-GFP or LAMP1-GFP on Ca^2+^ responses to TPC2-A1-N (30 µM) and TPC2-A1-P (60 µM). Cells were transiently transfected and segregated according to whether they were GFP-positive or -negative. Results are shown as responses of individual cells from a typical field of view (**h**, **k**) or as mean ± sem from 3 experiments (**i**, **l**). Pooled data quantifying the peak change in ratio are shown in **j** and **m**. Epifluorescence images of GFP and Fura-2 (380 nm excitation) from a typical field of view showing transfected (+) and non-transfected (–) cells. **p = 0.008, n.s. not significant (Unpaired t-test, two-tailed). **n** CRISPR targeting strategy for knockout of TPC2 in SK-MEL-5 cells (top) and qPCR validation (bottom) presented as mean ± sem from 3 experiments. ****p < 0.0001 (Unpaired t-test, two-tailed). **o**, **p** Effect of TPC2-A1-N (10 µM or 30 µM) and/or TPC2-A1-P (30 µM or 60 µM) on cytosolic Ca^2+^ in wild-type (WT) and TPC2 knockout (KO) SK-MEL-5 cells (**o**). Dare are mean ± s.e.m from 3 to 19 experiments. Pooled data quantifying the peak change in ratio in response to the indicated agonist concentration are shown in **p**. *p = 0.01, **p = 0.005, n.s. not significant (Unpaired t-test, two-tailed); ****p < 0.0001 (Mann-Whitney test, two-tailed). Source data are provided as a Source Data file.

In the next series of experiments, we examined the consequences of activating endogenous TPC2 on cellular Ca^2+^ signals. As shown in Fig. 3a, TPC2-A1-N induced a detectable Ca^2+^ response in single Fura-2 labelled HeLa cells. But the response was sluggish and modest in amplitude relative to responses in cells overexpressing TPC2 (Fig. 1b). TPC2-A1-P however had little detectable effect (Fig. 3a). Co-addition of the agonists induced robust Ca^2+^ responses (Fig. 3a, b), consistent with the synergistic activation of recombinant TPC2. The effect was particularly pronounced when the cells were simulated with TPC2-A1-P prior to TPC2-A1-N (Fig. 3c, d).

To establish specificity, we took three approaches. First, we examined the effects of inactive chemical analogues of TPC2-A1-N and TPC2-A1-P as negative controls (Fig. 3e). In the TPC2-A1-N analogue SGA-10, two chlorine residues at one of the benzenoid rings were replaced by hydrogen atoms (Fig. 3e)^24^. As shown in Fig. 3f, g, SGA-10 failed to evoke Ca^2+^ signals in HeLa cells consistent with a selective effect of the parent compound on TPC2. When combined with TPC2-A1-P, there was a small increase in the Ca^2+^ signal. In the TPC2-A1-P analogue SGA-153, the cyclohexylmethyl residue at the pyrrole nitrogen was replaced by an isopropyl residue (Fig. 3e)^24^. Like TPC2-A1-P, SGA-153 had little effect on cytosolic Ca^2+^ levels (Fig. 3f, g). But in contrast to TPC2-A1-P, SGA-153 only moderately potentiated the response to TPC2-A1-N (Fig. 3f, g), again attesting to specificity. In the second approach, we examined the effect of pore-dead TPC2 on agonist-evoked Ca^2+^ signals. Expression of TPC2^L265P^ significantly reduced the response to the agonist combination compared to cells expressing LAMP1 (Fig. 3h, i) or un-transfected cells (Fig. 3k–l). These results (summarized in Fig. 3j and m) are consistent with the pore mutant acting in a dominant-negative manner^28^. Third, we used CRISPR-Cas9 to knockout TPC2. For these experiments, we targeted TPC2 in SK-MEL-5 cells which express high levels of TPC2^29^. TPC2 depletion reduced TPC2 transcript levels by >90% (Fig. 3n) and reduced agonist-evoked currents (Supplementary Fig. 2). In control cells, the TPC2-A1-N and TPC2-A1-P combination again evoked robust Ca^2+^ signals (Fig. 3o). The initial rate of rise of these signals (Fig. 3o) was ~2-fold faster than those evoked in HeLa cells (Fig. 3a). Upon TPC2 targeting, the response to the agonist combination was substantially reduced (Fig. 3o). Similar inhibitory effects of TPC2 depletion were observed using a fixed concentration of TPC2-A1-N and increasing concentrations of TPC2-A1-P (Fig. 3p).

Taken together, these chemical, molecular and genetic analyses indicate that co-activation of endogenous TPC2 synergistically activate Ca^2+^ fluxes.

### Co-activation of TPC2 regulates lysosomal function

**Fig. 4:**
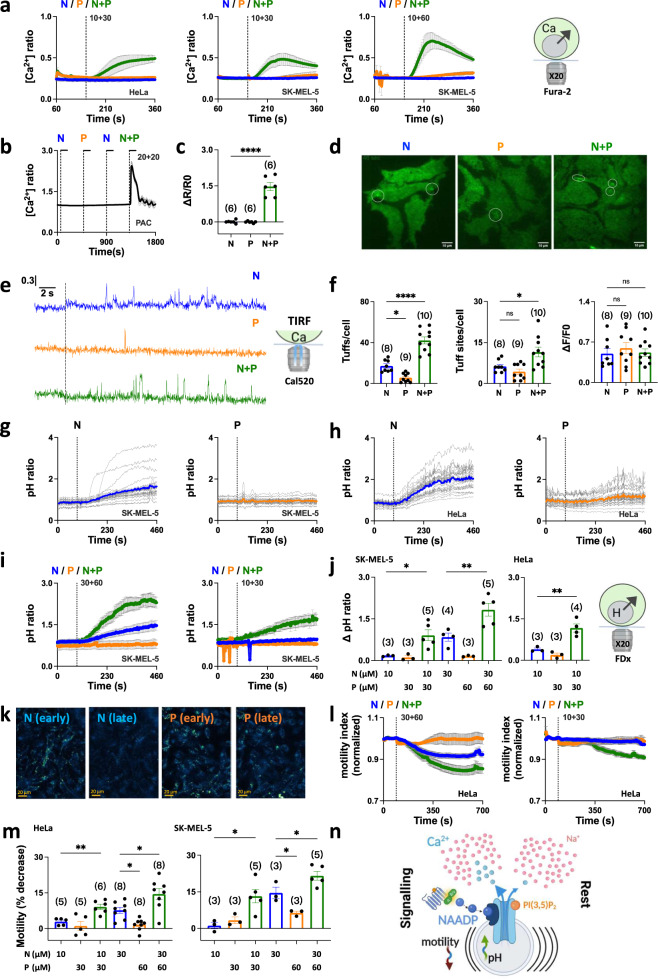
Co-activation of TPC2 regulates lysosomal function. **a** Effect of subthreshold concentration of TPC2-A1-N (10 µM), the indicated concentration of TPC2-A1-P or a combination on Ca^2+^ levels of individual HeLa or SK-MEL-5 cells loaded with Fura-2. Data are presented as mean ± sem from 3 to 9 experiments. **b**, **c** Effect of subthreshold concentration of TPC2-A1-N (20 µM), TPC2-A1-P (20 µM) or a combination of the two on Ca^2+^ levels of individual primary mouse pancreatic acinar cells loaded with Fura-2 **b**. Each trace is the normalized fluorescence ratio response of a single cell imaged from a typical field of view. The thicker trace is the average of the population. Pooled data (mean ± sem) quantifying the peak change in ratio from 6 experiments are shown in **c**. ****p < 0.0001 (Unpaired t-test, two-tailed). **d**–**f** Effect of TPC2-A1-N (30 µM), TPC2-A1-P (30 µM) or a combination of the two on subcellular Ca^2+^ levels of individual HEK cells loaded with Cal-520. Typical TIRF images with elementary events highlighted by circles are shown in **d**. Representative time courses of fluorescence changes from the centre of single tuff sites (1 × 1 μm) in response to the indicated agent. **e** Pooled data (mean ± sem) quantifying the number of events and sites detected per cell, and the peak response from 8 to 10 experiments are shown in **f**. *p < 0.05, ****p < 0.0001, n.s. not significant (One-way ANOVA followed by Dunnett’s post hoc test). **g**, **h** Effect of TPC2-A1-N (30 µM) and TPC2-A1-P (60 µM) on lysosomal pH of individual SK-MEL-5 (**g**) or HeLa (**h**) cells loaded with fluorescein-dextran (FDx). Each trace is the fluorescence ratio response of a single cell imaged from a typical field of view. The thicker trace is the average of the population. **i, j**, Effect of the indicated combinations of TPC2-A1-N and TPC2-A1-P on lysosomal pH of SK-MEL-5 cells **i**. Data are presented as mean ± sem from 3 to 5 experiments. Pooled data quantifying the peak change in ratio from multiple experiments using the indicated cell type and agonist combination are shown in **j**. *p = 0.02, **p < 0.01 (One-way ANOVA followed by Dunnett’s post hoc test). **k**–**m** Effect of TPC2-A1-N and TPC2-A1-P on lysosomal motility. Images show maximum projections of motility calculated from differences in pixel-wise intensity on a frame-by-frame basis over an early (120–240 s) and late (570−690 s) period following addition of TPC2-A1-N (30 µM) or TPC2-A1-P (60 µM) to HeLa cells **k**. Intense signals represent large changes over time equating to more lysosome movement. Full time-courses presented as mean ± sem from 3 to 8 experiments in response to the indicated agonist combination are shown in **l**. Pooled data quantifying motility at 700 s from multiple experiments using the indicated cell type and agonist combination **m**. *p < 0.05, **p = 0.008 (One-way ANOVA followed by Dunnett’s post hoc test). **n** Model showing that NAADP and PI(3,5)P_2_ work in a synergistic manner to selectively optimize Ca^2+^ signalling from lysosomes to regulate lysosomal pH and motility leaving Na^+^ signals unperturbed. Source data are provided as a Source Data file.

In the final set of experiments, we examined the functional impact of TPC2 co-activation. Lysosomes have long been thought to generate local Ca^2+^ signals during NAADP-mediated signaling events that ‘trigger’ Ca^2+^ release from the neighboring ER resulting in global Ca^2+^ signals^30^. These events, however, have been difficult to resolve. To investigate this putative coupling event, we first examined the effects of TPC2-A1-P on a sub threshold concentration of TPC2-A1-N which alone fail to evoke detectable Ca^2+^ signals. As shown in Fig. 4a, TPC2-A1-N (10 µM) was without effect on cytosolic Ca^2+^ levels in both HeLa cells and SK-MEL-5 cells. However, co-activation with TPC2-A1-P resulted in robust signals particularly in SK-MEL-5 cells (Fig. 4a) but less so in TPC2 KO cells (Fig. 3p). We also examined the effects of TPC2 agonists in primary pancreatic acinar cells (Supplementary Fig. 3). These cells were the first mammalian cells in which the effects of NAADP were characterized^31^. As shown in Fig. 4b, c, at a low concentration (20 µM) neither TPC2-A1-N nor TPC2-A1-P alone affected cytosolic Ca^2+^ levels. But again, the combination elicited a robust response.

To further examine the local-global transition, we used high resolution TIRF microscopy to define the spatio-temporal nature of the Ca^2+^ signals mediated by TPC2. These experiments were performed in HEK-293 cells in which local IP_3_-mediated Ca^2+^ signaling events have been extensively characterized^32,33^. As shown in Fig. 4d–f and Supplementary Movie 1, TPC2-A1-N evoked highly localized Ca^2+^ signals somewhat reminiscent of fundamental Ca^2+^ signals evoked by IP_3_ receptors termed ‘puffs’. We therefore refer to these events as ‘tuffs’, reflecting their origin (TPC2), their form (puff-like) and lack of ease to capture (tough; homophone). Tuffs were also resolved in response to TPC2-A1-P but these events were less frequent (Fig. 4e, Supplementary Movie 2). Co-activation of TPC2 substantially increased tuff frequency without affecting tuff amplitude (Fig. 4e, f, Supplementary Movie 3). Tuffs evoked by these means were also kinetically similar to those evoked by TPC2-A1-N and TPC2-A1-P alone, with comparable rise and fall times (Supplementary Fig. 4). However, there was a significant increase in the number of sites from which tuffs originated when TPC2 was co-activated by its ligands (Fig. 4f).

The pH of lysosomes is key to their degradative function and under acute control by NAADP and direct TPC2 activation^24,34–36^. We therefore examined the consequences of the agonist combinations on lysosomal pH. pH was measured ratiometrically with endocytosed fluorescein dextran. TPC2-A1-N increased lysosomal pH in SK-MEL-5 cells whereas TPC2-A1-P did not (Fig. 4g). Similar results were obtained in HeLa cells (Fig. 4h, Supplementary Movie 4). As shown in Fig. 4i, j, lysosomal pH responses upon co-activation of TPC2 were substantially larger in both cell types. This was particularly striking at low concentrations of TPC2-A1-N which alone induced small pH responses (summarized in Fig. 4j). Attempts to compare pH responses in TPC2 knockout SK-MEL-5 cells were confounded by differential uptake, distribution and baseline stability of fluorescein dextran (Supplementary Fig. 5).

Lysosomes are dynamic organelles that interact with the cytoskeleton^37^. The consequences of native TPC2 activation on lysosome motility was therefore also examined. As shown in Fig. 4k, TPC2-A1-N but not TPC2-A1-P reduced lysosome motility in HeLa cells (Supplementary Movie 4). To quantify motility, we computed the mean of pixel-wise absolute differences in lysosome labelling from timelapses between each time point and the next. The resulting profiles revealed that lysosome motility was slowed by TPC2-A1-N in a time-dependent manner (Fig. 4l). As with the pH responses (Fig. 4i, j), there was clear synergism between the agonists such that the agonist combination caused a larger change in motility than either of the agonists alone (Fig. 4l–m). And again, marked synergism was apparent upon near-threshold stimulation with TPC2-A1-N (Fig. 4l–m). Similar regulation of lysosome dynamics by TPC2 was evident in SK-MEL-5 cells (Fig. 4m).

In sum, co-activation of TPC2 globalizes lysosomal-derived Ca^2+^ signals, regulating lysosomal pH and motility.

## Discussion

TPC2 functions as a Ca^2+^-permeable, non-selective cation channel when activated by the Ca^2+^ mobilizing messenger NAADP and as a Na^+^-selective channel when activated by the phosphoinositide PI(3,5)P_2_. Here we show that despite radically different effects of TPC2 agonists on channel behavior, they work synergistically to selectively control Ca^2+^ flux and lysosome activity (Fig. 4n).

Whereas Ca^2+^ fluxes and currents through TPC2 upon co-activation were dramatically enhanced, Na^+^ flux and currents were largely unaltered (Figs. 1–2). Such a selective effect is remarkable considering that both ions share the same permeation pathway. Mechanistically, our previous work revealed that the ion selectivity of TPC2 is agonist-dependent allowing TPC2 to toggle between a selective (Na^+^) and a non-selective (Ca^2+^-permeable) state^24^. But increased Ca^2+^ currents through TPC2 reported here could not be explained in full by changes in ion selectivity because fully liganded TPCs had either the same or lower relative permeabilities to Ca^2+^ versus Na^+^ compared to TPC2 activated by NAADP (or its mimetic) alone. We speculate that the ensemble current as well as ion selectivity of TPC2 can be independently regulated by its ligands, the interplay of which will dictate net flux from the lysosome. In our experiments, NAADP was a less consistent activator of TPC2 compared to the other activators (Fig. 2). This likely reflects its indirect mechanism of action through NAADP-binding proteins^15,16,38^ which may differentially dissociate.

Functionally, we show that co-activation of endogenous TPC2 regulates several lysosomal activities (Fig. 4). Beyond their pH-dependent role in degradation, it is clear now that lysosomes are dynamic Ca^2+^ stores serving the cell in both ‘local’ mode to regulate membrane traffic and ‘global’ mode during signaling^39^. We succeeded in resolving tuffs, local TPC2-dependent Ca^2+^ signals (Fig. 4d–f). Intriguingly tuffs evoked by TPC2-A1-P although much less frequent than those evoked by TPC2-A1-N were indistinguishable in terms of their amplitudes and kinetics (Fig. 4e, f; Supplementary Fig. 4). We therefore predict that the unitary Ca^2+^ conductance of TPC2 is agonist-independent and that the differing Ca^2+^ permeabilities are due to changes in open probability. Of note, we found that the number of tuff sites increased when TPC2 was co-activated. These data indicate heterogeneity in agonist sensitivity of individual lysosomes and point to the existence of a population of normally ‘silent’ TPC2 channels. Thus, enhanced Ca^2+^ signaling upon TPC2 co-activation likely results in changes at both the molecular and organellar level.

Direct measurements of cellular NAADP show that it is a second messenger; its levels are low in resting cells but rise rapidly in response to a number of Ca^2+^ mobilizing stimuli^27^ often transiently^40^. PI(3,5)P_2_ is a low abundance phosphoinositide^23^. We mimicked signaling scenarios in an intact cell setting through sequential additions of TPC-A1-P and TPC2-A1-N (Figs. 1k–l, 3c, d). The resulting Ca^2+^ changes were robust and global in nature. PI(3,5)P_2_ levels are also under environmental control e.g. hypertonic shock in yeast^41^. And again, sequential activation of TPC2 by TPC2-A1-P after TPC2-A1-N revealed robust Ca^2+^ responses (Fig. 1m, n). One implication of this is that PI(3,5)P_2_ can (somewhat radically) be thought of as a Ca^2+^ mobilizing messenger in the presence of NAADP despite signaling through Na^+^ in its absence. But how widespread agonist-evoked production of PI(3,5)P_2_ in mammalian cells remains unclear. We therefore favour a model where PI(3,5)P_2_ sets the Ca^2+^ signaling capability of NAADP consistent with previous work showing NAADP-mediated Ca^2+^ signals are stimulated upon overexpression of PIKfyve and inhibited by PIKfyve inhibitors^42^. Regardless, TPC2 can be viewed as a coincidence detector able to tune its behavior depending on the relative levels of its activators. Although Ca^2+^ signals evoked by activation of endogenous TPC2 were attenuated by inactive TPC2 analogues, dominant negative TPC2 and TPC2 knock-out (Fig. 3), they were not abolished raising the possibility of some off-target effects of the agonist combination.

Beyond Ca^2+^, we found that both the acidity and motility of lysosomes were regulated by native TPC2 channels in an agonist-selective and synergistic way (Fig. 4g–m). The increase in pH might reflect permeability of TPC2 to H^+^^24^ and/or increases in luminal H^+^ buffering capacity coupled to cation release. Interestingly, TPC2 knockout also appeared to destabilise lysosomal pH in our hands (Supplementary Fig. 5) adding to the debate surrounding the role of TPC2 in regulating pH^7,20^. We speculate that the decrease in lysosome movement upon TPC2 activation facilitates inter-organellar communication with the ER, much like that reported for mitochondria during ER-mitochondria Ca^2+^ transfer^43^. With Na^+^ fluxes unperturbed, Na^+^-dependent functions of TPCs e.g., regulating membrane potential^20^ or osmotic balance^44^ likely remain intact during activation. In this way, segregated fluxes through TPC2 selectively facilitate lysosomal Ca^2+^ signaling.

## Methods

### Cells

HeLa cells, HEK-293 cells (wild type^32^ or stably expressing human TPC2^L11/L12A^-mRFP^45^ or TPC2-YFP^46^) and SK-MEL-5 cells (wild type or TPC2 knockout) were maintained in Dulbecco’s Modified Eagle Medium (DMEM), supplemented with 10% (v/v) Fetal Bovine Serum (FBS), 100 μg/mL streptomycin and 100 units/mL penicillin (all from Invitrogen) at 37 °C in a humidified atmosphere with 5% CO_2_. These lines are not commonly misidentified. Cells were passaged with trypsin. Cells were plated onto coverslips coated with poly-L-lysine (20–100 μg/mL, Sigma) for epifluorescence imaging and electrophysiology or with poly-D-lysine (100 μg/mL, Sigma) for TIRF imaging. For vacuolar patch clamp measurements, cells were treated with apilimod (1 µM) for 14 h to 18 h to enlarge endo-lysosomal organelles. For plate reading, cells were plated onto opaque-walled 96 well microplates (Corning).

Pancreatic acinar cells were obtained from male, 8–12 weeks old C57BL/6 J mice (Jackson Laboratories) housed at 22 ± 1 °C, with humidity not less than 30% on a 12 h light and dark cycle following CO_2_ asphyxiation and cervical dislocation according to The University of Rochester’s University Committee on Animal Resource (Protocol UCAR-2001-214E). Pancreata were enzymatically digested with type II collagenase (Sigma) in oxygenated DMEM (Invitrogen) with 0.1% bovine serum albumin (BSA) and 1 mg/mL soybean trypsin inhibitor for 30 min at 37 °C and 70 RPM in a shaking water bath. Cells were gently triturated to break up acinar clumps. Acini were then filtered through nylon mesh with a pore size of 100 µm, centrifuged at 75 × *g* through 4% BSA in DMEM, and resuspended in DMEM with 1% BSA.

### Chemicals

TPC2-A1-N, TPC2-A1-P, SGA-10, and SGA-153 were synthesized as described previously^24^. For some experiments, TPC2-A1-N and TPC2-A1-P were purchased from MedChem Express.

### Plasmids

Plasmids used were TPC2-GCaMP6s^24^, TPC2^L265P^-GCaMP6s^24^, LAMP1-GFP^47^, TPC2^L265P^-GFP^25^, TPC2^L11A/L12A^-GFP^25^, TPC2^L11A/L12A/K204A^-GFP^24^ and TPC2^L11A/L12A/L265P^-GFP^24^. HeLa cells were transiently transfected with plasmids 18-26 hrs prior to imaging, using lipofectamine^TM^ 2000 (from Invitrogen) according to the manufacturer’s instructions.

### TPC2 knockout

TPC2 knockout was created in the SK-MEL-5 melanoma cell line. Exon 3 in *TPCN2* was targeted, by designing guide RNAs in Intron 2/3 and Intron 3/4 (Supplementary Fig. 2). This strategy led to a frameshift mutation, rendering nonsense protein translations of TPC2 and reduced agonist-evoked vacuolar currents (Supplementary Fig. 2). Protocols were as previously described for targeting the *MCOLN1* gene in^48^ and will be described in full elsewhere.

### Single cell epifluorescence microscopy

Cytosolic Ca^2+^, cytosolic Na^+^ and lysosomal pH were measured at the single cell level using fluorescent probes.

For HeLa cells, HEK-293 cells stably expressing human TPC2^L11/L12A^ and SK-MEL-5 cells, cytosolic Ca^2+^ was measured using the genetically-encoded Ca^2+^ indicator GCaMP6s fused to the C-terminus of TPC2 or the fluorescent dyes, Fura-2 (from Biotium) and Fura-FF (from Cayman Chemical). Ca^2+^ imaging experiments were performed at room temperature in HEPES-buffered saline (HBS1) containing 10 mM NaHEPES, 1.25 mM KH_2_PO_4_, 2 mM MgSO_4_, 3 mM KCl, 156 mM NaCl, 2 mM CaCl_2_ and 10 mM glucose (pH 7.4; all from Sigma-Aldrich). For dye loading, cells were incubated with Fura-2 AM or Fura-FF AM (2.5 µM) and 0.005% (v/v) pluronic acid (from Invitrogen) for 1 h in HBS. Where indicated, some experiments were performed in nominally Ca^2+^-free HBS where CaCl_2_ was omitted and the cells were stimulated with ionomycin (Ca^2+^ salt, Cayman Chemical) toward the end of recording period.

For pancreatic acinar cells, cytosolic Ca^2+^ was measured using Fura-2. Ca^2+^ imaging experiments were performed at room temperature in HEPES-buffered saline (HBS2) containing 137 mM NaCl, 0.56 mM MgCl_2_, 4.7 mM KCl, 1 mM Na_2_HPO_4_, 10 mM HEPES, 5.5 mM glucose, and 1.26 mM CaCl_2_ (pH 7.4). Cells were incubated with Fura-2-AM (5 µM; Thermofisher) in HBS2 supplemented with 1% BSA for 30 min. Fura-2 loaded cells were adhered to a Cell-Tak (Corning)-coated glass coverslip in a Warner perfusion chamber and perfused with HBS2.

Cytosolic Na^+^ in HEK cells stably expressing TPC2^L11/L12A^ was measured using the fluorescent Na^+^ indicator SBFI. Na^+^ imaging experiments were performed at room temperature in HBS. Cells were incubated with SBFI AM (5 µM) and 0.005% (v/v) pluronic acid (both from Invitrogen) for 1 h in HBS. Where indicated, some experiments were performed in low Na^+^ HBS where NaCI was replaced by NMDG (Sigma).

Lysosomal pH in HeLa and SK-MEL-5 cells was measured using fluorescein in HBS at room temperature. Cells were loaded with fluorescein-dextran (0.1 mg/mL; MW 10,000; from Invitrogen) by endocytosis overnight in culture followed by up to 10 hrs chasing period in dextran-free culture medium.

After transfection and/or dye loading, cells were washed in HBS and were subsequently mounted in a 1 mL imaging chamber (Biosciences Tools) for microscopy. Epifluorescence images were acquired every 3 s. For Fura-2, Fura-FF, SBFI and some GCaMP6s measurements, images were captured with a cooled coupled device camera (TILL photonics) attached to an Olympus IX71 inverted fluorescence microscope fitted with a monochromatic light source under the control of TillVision 4.0 software. Fura-2, Fura-FF, and SBFI were excited at 340/380 nm and emitted fluorescence was captured using a 440 nm long-pass filter at 20× magnification. GCaMP6s was excited at 470 nm and emitted fluorescence was captured using a 515 nm long-pass filter with a 40× objective.

For fluorescein measurements and other GCaMP6s measurements, images were captured using a Megapixel monochrome cooled coupled device camera attached to an Olympus IX73 inverted fluorescence microscope fitted with a CoolLED multiple wavelength LED source under the control of MetaFluor 7.10.3.279 software. Fluorescein was excited at 490 nm/405 nm and emitted fluorescence was captured using a 510 nm long-pass filter at 20× or 40× magnification. GCaMP6s was excited at 470 nm and emitted fluorescence was captured using a 510 nm long-pass filter with a 20× objective.

For Fura-2 measurements in pancreatic acinar cells, imaging was performed using an inverted Olympus IX-71 microscope through a 40× oil immersion objective lens (N.A. = 1.35). Cells were excited alternately with UV at wavelengths of 340 and 380 nm using a monochromator-based illumination system (TILL Photonics), and the emission at 510 nm was captured using a Sensicam QE camera under the control of TillVision 4.0 software.

### Population-based cytosolic Ca^2+^ measurements

Cytosolic Ca^2+^ in populations of HEK stably expressing TPC2^L11/L12A^ was measured using Fura-2 and a fluorescence plate reader (Clariostar, BMG Labtech) under the control of Mars 3.42 R3 software. Cells were incubated with Fura-2 AM (2.5 µM) and 0.005% (v/v) pluronic acid (from Invitrogen) for 1 h in HBS. A single measurement comprised 16 flashes at 335 nm and 380 nm (each at 8 nm bandpass) while recording fluorescence at 520 nm (90 nm bandpass). Measurements were repeated on an individual well at 40 s intervals with 15 wells being recorded in parallel using “plate mode”. Defined volumes of TPCA1-N and TPCA1-P, each at 210 µM, were added simultaneously through two independent injector needles to achieve the indicated final concentrations. Background fluorescence was measured from wells containing cells that were incubated with HBS without Fura-2.

### Subcellular cytosolic Ca^2+^ measurements

Elementary cytosolic Ca^2+^ signals in wild-type HEK-293 cells were measured using Cal-520 and TIRF microscopy. Prior to imaging, the cells were washed three times with HBS2. The cells were subsequently incubated with Cal520-AM (5 µM; AAT Bioquest #21130) and ci-IP_3_/PM (0.5 μM, Tocris #6210) in HBS2 supplemented with 0.01% BSA in dark at room temperature. After 1-h incubation, the cells were washed three times with HBS2 and incubated in HBS2 containing EGTA-AM (5 μM, Invitrogen #E1219). After 45 min incubation, the media was replaced with fresh HBS2 and incubated for additional 30 min at room temperature to allow for de-esterification of loaded reagents^49^.

Following loading, the coverslip was mounted in a chamber and imaged using an Olympus IX83 inverted total internal reflection fluorescence (TIRF) microscopee equipped with an oil-immersion PLAPO OTIRFM 60× objective lens/1.45 numerical aperture. The cells were illuminated using a 488 nm laser to excite Cal-520 and the emitted fluorescence was collected through a band-pass filter by a Hamamatsu ORCA-Fusion CMOS camera. The angle of the excitation beam was adjusted to achieve TIRF with a penetration depth of ~140 nm. Images were captured from a field of view by directly streaming into RAM. TIRF images were captured using 2 × 2-pixel binning (216 nm/pixel) from equal field of views for HEK-293 cells at a rate of ~50 frames per second. Agonists were applied directly to the imaging chamber.

After visualizing images with the cellSens [Ver.2.3] life science imaging software (Olympus), images were exported as vsi files as described in^50^ The vsi files were converted to tif files using ImageJ 1.53f51and further processed using FLIKA (Ver 1), a Python programming-based tool for image processing^51^. From each recording, 200 frames (~4 s) before agonist addition were averaged to obtain a ratio image stack (F/F0) and standard deviation for each pixel for recording up to 30 s following photolysis. The image stack was Gaussian-filtered, and pixels that exceeded a critical value (0.8 for our analysis) were located. The ‘Detect-puffs’ plug-in was utilized to detect the number of clusters, number of events, amplitudes and durations of localized Ca^2+^ signals from equal areas across different conditions from individual cells. All the events identified automatically by the algorithm were manually confirmed before further analysis^32,52^.

### Cell surface patch-clamp measurements

Currents were recorded in the inside-out configuration from macropatches excised from the plasma membrane of HEK-293 cells stably expressing TPC2^L11/L12A^. Data were acquired using an AxoPatch 200 B amplifier (Molecular Devices) and pClamp10.2 suite (Molecular Devices). Records were filtered at 2 kHz and digitized at 10 kHz using Digidata 1440 A (Molecular Devices). ClampFit 10.2 was used for offline analysis of data. Currents were evoked by voltage ramps from −100 mV to +100 mV over 400 ms repeated at 5 s intervals from a holding potential of 0 mV.

Patch-pipettes were pulled from thick-walled, filamented borosilicate glass capillaries (Sutter Instrument) using Narishige PC-10 vertical puller, fire polished using a Narishige MF-830 microforge (Digitimer Ltd.). The pipette (luminal) solution contained (in mM): 105 CaCl_2_, 5 HEPES, 5 MES (pH adjusted to 4.6 using MSA). The bath (cytosolic) solution contained (mM): 160 NaCl and 5 HEPES (pH adjusted to 7.2 using NaOH). Pipettes had a resistance of 1–3 MΩ when filled with the pipette solution. Liquid junction potentials were estimated using pClamp 10 and corrected as described previously^53^.

TPC2-A1-N, TPC2-A1-P, PI(3,5)P_2_ (diC8 form; Echelon Biosciences), and NAADP (Tocris) were applied to the bath solution of excised macropatches via an 8-channel pressurized perfusion system controlled by ValveLink 8.2 controller (AutoMate Scientific). All electrophysiological recordings were made at room temperature (21–23 °C).

The permeability ratio (P_Ca_/P_Na_) was calculated from the reversal potential according to^54^:

$$\frac{{P}_{{Ca}}}{{P}_{{Na}}}=\frac{{\gamma }_{{Na}}}{{\gamma }_{{Ca}}} * \,\frac{{\left[{Na}\right]}_{i}}{{4\left[{Ca}\right]}_{o}} * {{\exp }}^{\left({E}_{{rev}}\frac{F}{{RT}}\right)} * ({{\exp }}^{\left({E}_{{rev}}\frac{F}{{RT}}\right)}+1)$$

where *P*_*Ca*_ = Ca^2+^ permeability; *P*_*Na*_ = Na^+^ permeability; ${\gamma }_{{Ca}}$ = Ca^2+^ activity coefficient (0.52); ${\gamma }_{{Na}}$= Na^+^ activity coefficient (0.75); [Ca]_o_ = concentration of Ca^2+^ in the lumen; [Na]_i_ = concentration of Na^+^ in the cytosol; ${E}_{{rev}}$ = reversal potential; F—Faraday’s constant, R—gas constant; T—absolute temperature.

### Vacuolar patch-clamp measurements

Currents were recorded in the whole-vacuole configuration from enlarged lysosomes manually excised from HEK-293 cells stably expressing TPC2 as described in ref. 55. Data were acquired, digitized (40 kHz) and filtered (2.9 kHz) using an EPC-10 amplifier and PatchMaster software v2x90.4 (both HEKA, Lambrecht, Germany). During each recording, fast and slow capacitive transients were cancelled by amplifier compensation circuit. Currents were evoked by voltage ramps from −100 mV to +100 mV repeated at 5 s intervals from a holding potential of 0 mV and normalized to organelle size.

Patch pipettes were pulled from borosilicate glass and polished to resistances in the range of 8–11 MΩ. Liquid junction potential was corrected as described^55^. Pipette and bath solutions were the same composition as those for the macropatch recordings.

TPC2-A1-N, TPC2-A1-P, PI(3,5)P_2_ (diC8 form; Echelon Biosciences) and NAADP (Bio-Techne) were applied by complete exchange of the cytoplasmic solution. All compounds were freshly diluted before experimentation.

### Lysosomal motility measurements

Lysosome motility was calculated from the images acquired for pH measurements. Cell-free areas were discarded by computing local standard deviations across the image and thresholding the result. Changes in intensity at 490 nm over time were normalized by dividing images by their mean intensity at each time point. Motility was quantified as the mean of pixel-wise absolute differences in normalized intensity between each time point and the next (3 s intervals). To attain robustness to artifacts such as a local loss of poorly attached cells upon agonist addition, images were split into 25 (5 × 5) equally sized chunks and those with cell coverage <1/3 anywhere along time course were removed. Motility was quantified in each remaining chunk independently and the resulting measures were combined by taking the median. A 1d median filter was applied to the resulting motility time profiles. Motility measures were normalized to the baseline prior to agonist addition.

### Statistics

Parametric tests were performed using a paired t-test, unpaired t-test or one-way ANOVA. Non-parametric tests were performed using Kruskal-Wallis or Mann-Whitney analysis, respectively. All data were analyzed using Prism 9 (GraphPad Software). *p < 0.05 **p < 0.01 ***p < 0.001 ****p < 0.0001.

All cartoons from Figs. 1–4 and Supplementary Fig. 2 and 5 were created with BioRender.

### Reporting summary

Further information on research design is available in the Nature Research Reporting Summary linked to this article.

## Supplementary information

Supplementary Information

Description of Additional Supplementary Files

Supplementary Movie 1

Supplementary Movie 2

Supplementary Movie 3

Supplementary Movie 4

Reporting Summary

## Data Availability

The source data underlying Figs. 1d, f, h, l, j, n, p, r, 2b, f, h, j, l, m, n, 3b, d, g, j, m, n, p, 4c, f, j, m, Supplementary Figs. 1b, d, f, h, 3b, 4, 5b are provided as a Source Data file. Source data are provided with this paper.

## Source data

Source Data
