# Supplementary material for: Segregated cation flux by TPC2 biases Ca2+ signaling through lysosomes
Source: Nat Commun. 2022 Aug 2;13:4481. doi: 10.1038/s41467-022-31959-0 (PMC9346130; doi:10.1038/s41467-022-31959-0)
Supplement: Supplementary file 1 — Supplementary Information [file 41467_2022_31959_MOESM1_ESM.pdf]

Supplementary information

Segregated cation flux by TPC2 biases Ca^{2+} signaling through lysosomes.

Yu Yuan[1], Dawid Jaślan[2], Taufiq Rahman[3], Stephen R. Bolsover[1], Vikas Arige[4], Larry E. Wagner II[4], Carla Abrahamian[2], Rachel Tang[2], Marco Keller[5], Jonas Hartmann[1], Anna S. Rosato[2], Eva-Maria Weiden[2], Franz Bracher[5], David I. Yule[4], Christian Grimm[2,*] and Sandip Patel[1,*]

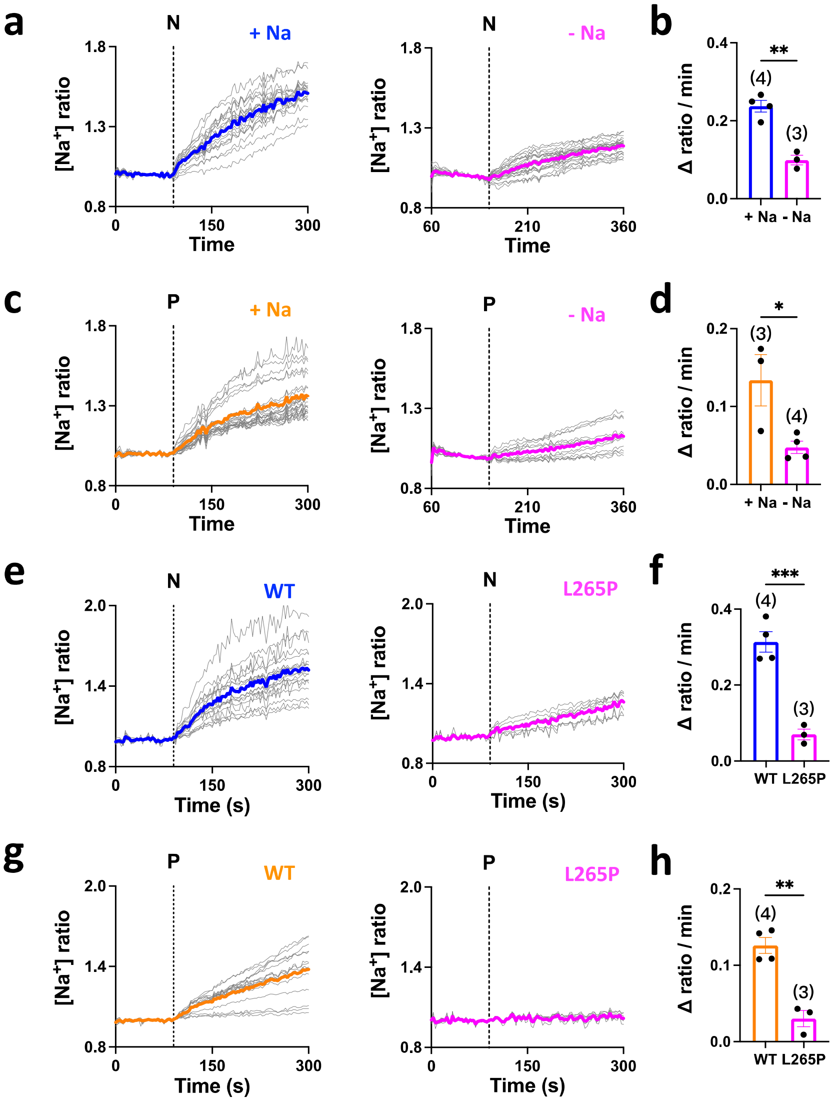

Supplementary Figure 1. Cell surface TPC2 mediates Na^+ influx.

a – d, Effect of TPC2-A1-N (30 μM, **a**) and TPC2-A1-P (30 μM, **c**) on Na^+ levels of individual SBFI-loaded HEK cells stably expressing TPC2$^{L11A/L12A}$. Experiments were performed in HBS (+Na^+) or HBS in which NaCl was replaced with NMDG (-Na^+). Each trace is the normalized fluorescence ratio response of a single cell imaged from a typical field of view. The thicker trace is the average of the population. Pooled data (mean ± s.e.m. from 3-4 experiments) quantifying the rate of Na^+ influx from multiple experiments is shown in **b** and **d**. *P=0.03, **P=0.001 (Unpaired t-test, two-tailed).

e – h, Effect of TPC2-A1-N (30 μM, **e**) and TPC2-A1-P (30 μM, **g**) on Na^+ levels of individual SBFI-loaded HeLa cells transiently expressing TPC2$^{L11A/L12A}$ or pore-dead TPC2$^{L11A/L12A/L265P}$. Experiments were performed in HBS. Each trace is the normalized fluorescence ratio response of a single cell imaged from a typical field of view. The thicker trace is the average of the population. Pooled data (mean ± s.e.m. from 3-4 experiments) quantifying the rate of Na^+ influx from multiple experiments are shown in **f** and **h**. **P=0.001, ***P=0.0008 (Unpaired t-test, two-tailed).

Source data are provided as a Source Data file.

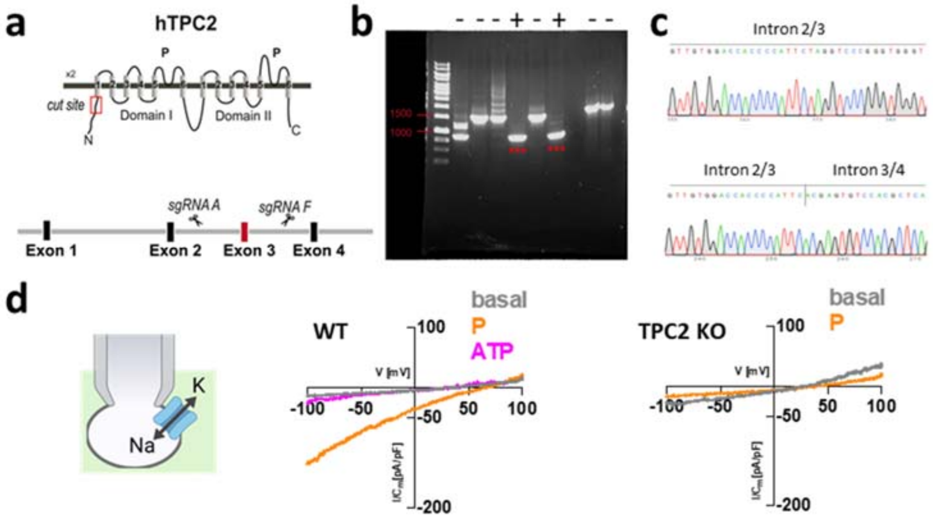

Supplementary Figure 2. Validation of TPC2 knockout cells.

a, CRISPR targeting strategy for knockout of TPC2 in SK-MEL-5 cells.

b, Genomic PCR analysis of independent clonal lines resulting in the identification of two positives that yielded products consistent with knockout (***). Expected sizes of the products were 1521 bp (wild type) and 831 bp (knockout).

c, Genomic sequencing wildtype (WT) and TPC2 knockout (KO) cells used in this study.

d, Effect of TPC2-A1-P (10 µM) and ATP (1 mM) on lysosomal currents recorded from wildtype and TPC2 knockout cells.

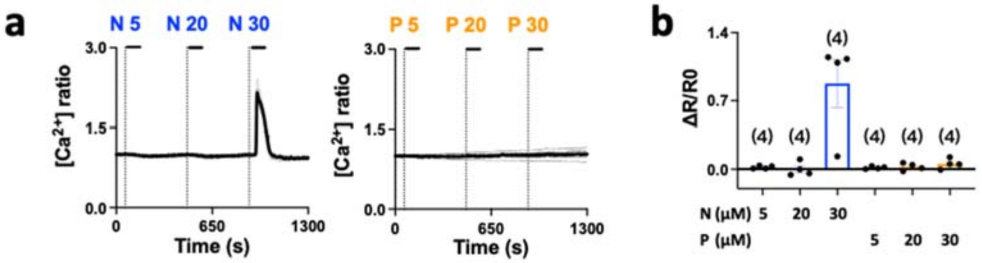

Supplementary Figure 3. Activation of native TPC2 evokes agonist-selective changes in Ca^{2+}.

a, Effect of increasing concentrations of TPC2-A1-N and TPC2-A1-P on Ca^{2+} levels of individual primary mouse pancreatic acinar cells loaded with Fura-2. Each trace is the normalized fluorescence ratio response of a single cell imaged from a typical field of view. The thicker trace is the average of the population.

b, Pooled data (mean ± s.e.m. from 4 experiments) quantifying the peak change in normalized ratio from multiple experiments.

Source data are provided as a Source Data file.

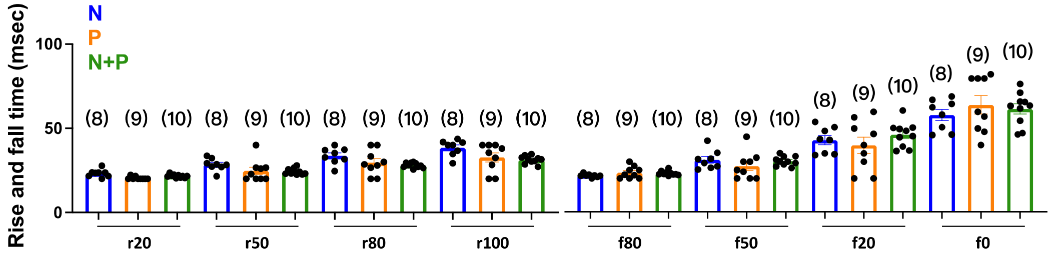

Supplementary Figure 4. Elementary TPC2-mediated Ca^{2+} signals are kinetically similar. Pooled data (mean ± s.e.m. from 8-10 experiments) showing the mean rise (r) and fall (f) times of tuffs recorded from individual HEK cells loaded with Cal-520. Data were calculated to the indicated normalized intensity level for an individual event.
Source data are provided as a Source Data file.

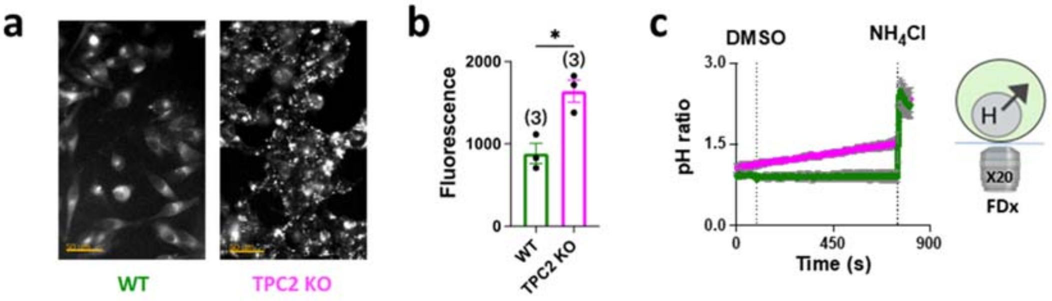

Supplementary Figure 5. TPC2 knockout confounds fluorescein dextran comparisons.
a, Representative epifluorescence images of wild-type and TPC2 knockout cells labelled with fluorescein dextran (excitation = 405 nm).
b, Pooled data (mean ± s.e.m. from 3 experiments) quantifying basal fluorescein dextran fluorescence. *P=0.01 (Unpaired t-test, two-tailed).
c, Ratiometric fluorescein dextran measurements in wild type and TPC2 knockout cells (mean ± s.e.m. from 3 experiments) stimulated with DMSO (0.1 %v/v) and NH_4Cl (5 mM).
Source data are provided as a Source Data file.
