# Supplementary material for: Segregated cation flux by TPC2 biases Ca2+ signaling through lysosomes
Source: Nat Commun. 2022 Aug 2;13:4481. doi: 10.1038/s41467-022-31959-0 (PMC9346130; doi:10.1038/s41467-022-31959-0)
Supplement: Supplementary file 2 — Description of Additional Supplementary Files [file 41467_2022_31959_MOESM2_ESM.pdf]

Description of Additional Supplementary Files

File Name: Supplementary Movie 1
Description: TIRF imaging showing the effect of TPC2-A1-N (30 µM) on subcellular Ca2+ levels of individual HEK cells loaded with Cal-520. # denotes agonist addition.

File Name: Supplementary Movie 2
Description: TIRF imaging showing the effect of TPC2-A1-P (30 µM) on subcellular Ca2+ levels of individual HEK cells loaded with Cal-520. # denotes agonist addition.

File Name: Supplementary Movie 3
Description: TIRF imaging showing the effect of a combination of TPC2-A1-N (30 µM) and TPC2-A1-P (30 µM) on subcellular Ca2+ levels of individual HEK cells loaded with Cal-520. # denotes agonist addition.

File Name: Supplementary Movie 4
Description: Epifluorescence imaging showing the effect of TPC2- A1-N (30 µM) and TPC2-A1- P (60 µM) on lysosome motility of HeLa cells loaded with Fluorescein-dextran. Agonist addition is denoted by N and P, respectively.
